# Prognostic Value of Combining Apelin-12 and Estimated Glomerular Filtration Rate in Patients with ST-Segment Elevation Myocardial Infarction

**DOI:** 10.1155/2022/2272928

**Published:** 2022-06-24

**Authors:** Yue Liu, Huasong Xia, Meng Li, Yi Chen, Yanqing Wu

**Affiliations:** Department of Cardiology, Second Affiliated Hospital of Nanchang University, No. 1 Mingde Road, Nanchang 330006, Jiangxi, China

## Abstract

**Background:**

Apelin-12 and estimated glomerular filtration rate (eGFR) are considered prognostic factors for ST-segment elevation myocardial infarction (STEMI). However, little is known about whether the combined use of these two biomarkers could enhance the prognostic value. This study aimed to investigate the utility of combining apelin-12 and eGFR for STEMI.

**Methods:**

Patients were divided into four groups based on median apelin-12 level and eGFR level: A: low apelin-12, low eGFR; B: low apelin-12, high eGFR; C: high apelin-12, low eGFR; and D: high apelin-12, high eGFR. The Cox regression was used to identify prognostic factors. The Kaplan–Meier and the receiver operating characteristic (ROC) curves were generated to evaluate the prognostic value of apelin-12 combined with eGFR in patients with STEMI.

**Results:**

Among 460 patients, 118 (25.7%) experienced major adverse cardiac events (MACEs) during the entire follow-up of 30 months. The Kaplan–Meier curve analysis revealed that group *D* had the best prognosis compared with the other three groups. The combination of apelin-12 and eGFR (area under the ROC curve (AUC), 0.699) enhanced the predictive value for MACE compared with either apelin-12 (AUC, 0.617) or eGFR (AUC, 0.596) alone. There was a negative association between apelin-12 and eGFR (*r* = −0.32, *p* < 0.001), while no association was observed between the Gensini score and apelin-12 or eGFR.

**Conclusions:**

This study suggests that both low apelin-12 (<0.76 ng/ml) and low eGFR (<94.06 mL/min/1.73 m^2^) are associated with poor prognosis in STEMI, indicating that the combination of apelin-12 and eGFR could enhance the prognostic value of patients with STEMI.

## 1. Introduction

ST-segment elevation myocardial infarction (STEMI), characterized by cardiomyocyte death due to persistent ischemia and hypoxia, is one of the most common and fatal diseases. Despite the decline in mortality rates in recent years [1], STEMI remains a significant threat to global health. According to the Jakarta Acute Coronary Syndrome Registry database [2], STEMI accounts for approximately 37% of acute coronary syndrome cases in developing countries, consistent with that reported in a previous study [3]. Although considerable advances have been achieved in reperfusion therapies and drug treatments, the prognosis of patients with STEMI remains far from satisfactory because many patients continue to experience complications and major adverse cardiac events (MACEs) after STEMI [4]. Therefore, it is necessary to identify risk factors associated with the prognosis of patients with STEMI.

Apelin-12, a member of the apelin family, is a ligand of the human orphan *G* protein-coupled receptor (APJ). It has been suggested that the apelin/APJ system plays a critical role in various diseases, including respiratory [5], gastrointestinal [6], and hepatic diseases [7]. Recently, apelin-12 has also been investigated in cardiovascular diseases. Liu and his teammates enrolled 120 patients with STEMI who underwent primary percutaneous coronary intervention (PCI) and were followed up for 12 months. The authors found that the incidence of MACE was significantly higher in patients with low apelin-12 levels than in those with high apelin-12 (*p* < 0.001) [8]. Another observational study suggested that the MACE rate was much lower in patients with an apelin-12 level >2.2 ng/mL [9]. The underlying cardioprotective mechanisms may be that the apelin system promotes vasodilation, reduces blood pressure in a nitric oxide-dependent way, increases cardiac contractility, and improves angiogenesis after myocardial infarction [10].

The estimated glomerular filtration (eGFR) is a useful indicator of quantifying renal function in clinical settings. The relationship between lower eGFR and poor outcomes in patients with STEMI has long been established. According to a study by Anavekar, for each reduction in eGFR by 10 units below 81.0 mL/min/1.73 m^2^, the risk of death and nonfatal cardiovascular outcomes is increased by 10% [11]. A previous study has suggested that apelin-12 has prognostic value in predicting short-term (during hospitalization) and long-term (2.5 years) MACE, especially in those with normal eGFR [12]. However, little is known about whether the combination of apelin-12 and eGFR has a better prognostic value than each biomarker alone.

Accordingly, this study aimed to determine whether the combination of apelin-12 and eGFR served as a better prognostic biomarker in patients with STEMI than either one individually.

## 2. Methods

**Figure 1:**
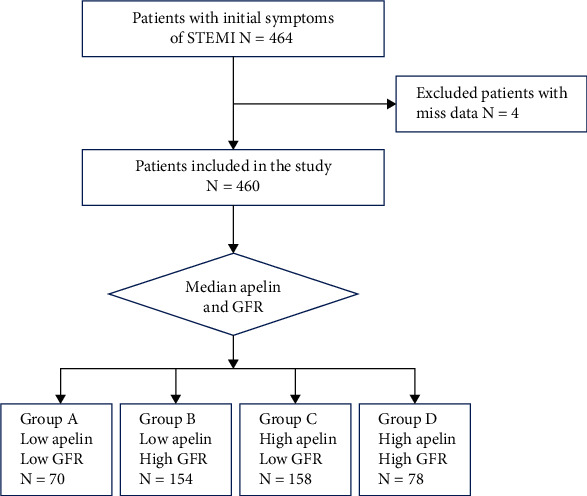
Flow chart of the study.

Clinical data of 464 patients with STEMI were downloaded from Dryad (https://doi.org/10.5061/dryad.pf56m), a publicly available database that houses a large quantity of datasets from published articles. After removing the data of four patients with missing information, this study ultimately included 460 patients with STEMI from the First People's Hospital of Taizhou, Zhejiang, China, who were admitted with STEMI symptoms between January 2010 and October 2014. The inclusion and exclusion criteria, therapy process of patients, and apelin-12 detection have been described previously [12]. The Gensini score was used to assess the severity and extent of coronary artery stenosis, and detailed information can be referred in the literature [13]. To compare prognosis, patients were initially divided into two groups according to the median levels of apelin-12 or eGFR. Further, patients were divided into four groups according to the median apelin-12 level and eGFR level: A (low apelin-12, low eGFR (*n* = 70)); B (low apelin-12, high eGFR (*n* = 154)); C (high apelin-12, low eGFR (*n* = 158)); and *D* (high apelin-12, high eGFR (*n* = 78)). The flow chart is shown in Figure 1. Because all patient data were anonymized and data analysis adhered to the Dryad database rules, neither ethics approval nor patient consent was required for this study. We verified that the sample size is sufficient using PASS software with an alpha of 0.05 and a power of 80%. We estimated a sample size of 308 patients, and our study included 460 patients.

The primary endpoint was the occurrence of MACE, defined as a composite of cardiac death, recurrent target vessel-related myocardial infarction, ischemia-driven target lesion revascularization, cardiogenic shock, or congestive heart failure. The eGFR was calculated using the equation published in the Modification of Diet in Renal Disease study equation [14]. More specifically,

(1) $$\begin{array}{c} \text{GFR}=186.3\times {\left(\text{Scr}\right)}^{\wedge }-1.154\times {\left(\text{age}\right)}^{\wedge }-0.203\times 0.742\left(\text{if female}\right). \end{array}$$

Hypertension was defined as blood pressure ≥140/90 mmHg and/or using antihypertensive drugs. T2DM was defined as FPG ≥7.0 mmol/L and/or on medication for T2DM.

All patients were followed up for 30 months or until the occurrence of MACE. Survival time was calculated from the date of PCI until an adverse event occurred.

For continuous variables, data are expressed as mean and standard deviation (SD) or median and interquartile range. Categorical variables are expressed as frequency (percentage). Differences between groups were compared using the one-way analysis of variance or the chi-square test.

The univariate Cox regression analysis was performed to identify potential prognostic factors. Variables with *p* < 0.05 except apelin-12 and eGFR were entered into the multivariate Cox regression analysis to identify the final factors. Because the variable “Group” was created by the levels of apelin and eGFR, putting them along with the group into the multivariate Cox regression will yield a collinearity effect. For survival analysis, the Kaplan–Meier curves were plotted, and the log-rank test was used for statistical analysis. To compare the discriminatory power between the combination of apelin-12 and eGFR and either apelin-12 or eGFR alone, receiver operating characteristic (ROC) curves were constructed. All analyses were performed using *R* software version 4.0.4 (R Foundation for Statistical Computing, Vienna, Austria). Differences with two-sided *p* < 0.05 were considered to be statistically significant.

## 3. Results

This study included 460 patients, 118 (25.7%) of whom experienced MACE during the follow-up period. As shown in Table 1, the mean (±SD) age of the patients was 62.9 ± 11.9 years, and the mean (±SD) systolic blood pressure was 132 ± 27.2 mmHg. The majority of patients (76.7%) were male. Except for creatinine level, no significant differences were observed among the four groups in terms of demographic information, medical history, and laboratory test results.

**Figure 2:**
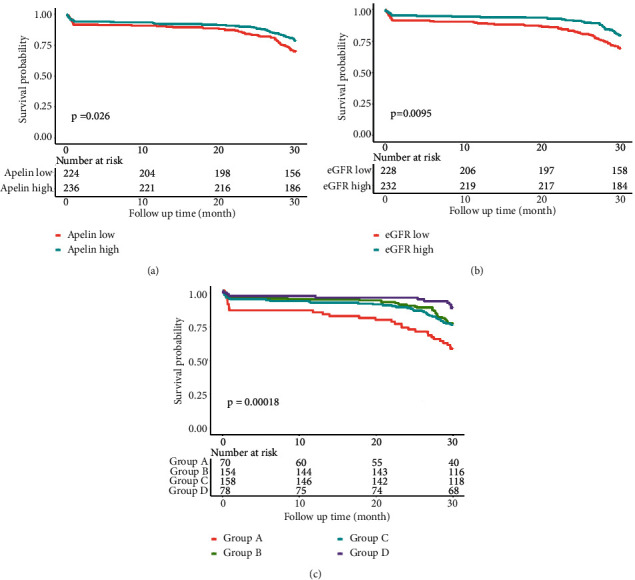
Kaplan–Meier curves for MACE. Kaplan–Meier curves for MACE based on apelin-12 (a), or eGFR (b), or the combination of apelin-12 and eGFR (c). eGFR: estimated glomerular filtration rate; MACE: major adverse cardiac events.

Patients were divided into two groups according to the median apelin-12 level or eGFR. As shown in Figure 2(a), patients with lower apelin-12 levels (<0.76 ng/mL) experienced a significantly higher occurrence of MACE than those with higher levels (*p*=0.026). Meanwhile, patients with low eGFR (<94.06 mL/min/1.73 m^2^) had a poorer prognosis than those with high eGFR (*p*=0.0095) (Figure 2(b)). As such, these results prompted us to ask whether the combination of apelin-12 and eGFR could serve as a better prognostic factor for patients with STMEI. Therefore, patients were divided into four groups based on the median apelin-12 level and eGFR. As shown in Figure 2(c), group *D* (high apelin-12, high eGFR) had the highest survival probability compared with group A (low apelin-12, low eGFR), suggesting that the combined use of apelin-12 and eGFR served as a better prognostic factor for patients with STEMI than the use of either biomarker alone.

**Figure 3:**
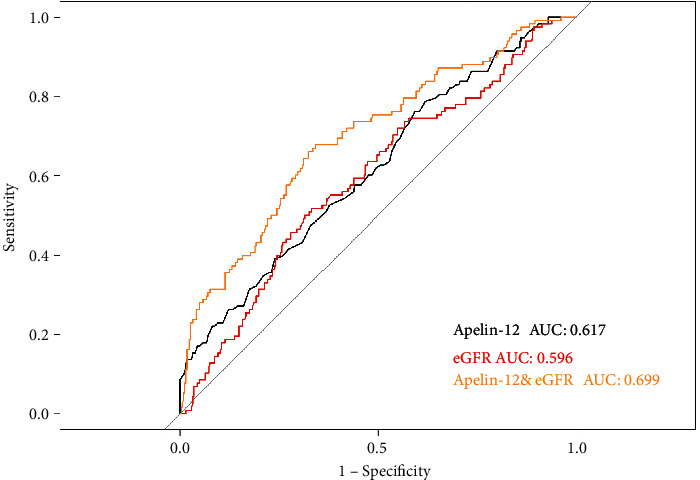
ROC curves for the combination of apelin-12 and eGFR, and either apelin-12 or eGFR alone. eGFR: estimated glomerular filtration rate; ROC: receiver operating characteristic.

The prognostic value of combining apelin-12 and eGFR was evaluated according to the area under the ROC curve (AUC). As shown in Figure 3, the combination of apelin-12 and eGFR (AUC 0.699) yielded a better predictive value for MACE than either apelin-12 (AUC: 0.617) or eGFR (AUC: 0.596) alone.

In univariate analyses, age, heart rate, anterior wall myocardial infarction (MI) history, Killip grade > I, apelin-12, hemoglobin, total cholesterol, high-density lipoprotein, eGFR, and pathological *Q* wave were identified as potential prognostic risk factors for MACE (Table 2). Multivariate analyses revealed that age (hazard ratio (HR): 1.03, 95% confidence interval (CI): 1.01 to 1.05; *p*=0.0008), anterior wall MI history (HR: 1.57, 95% CI: 1.06 to 2.32; *p*=0.0243), TC (HR: 1.23, 95% CI: 1.05 to 1.44; *p*=0.0121), and pathological *Q* wave (HR: 1.73, 95% CI: 1.18 to 2.53; *p*=0.0047) were associated with an increased risk of MACE. Compared with patients in group A, those in group B (HR: 0.6, 95% CI: 0.37 to 0.98; *p*=0.0399), group C (HR: 0.55, 95% CI: 0.34 to 0.89; *p*=0.0151), and group *D* (HR: 0.27, 95% CI: 0.13 to 0.57; *p*=0.0005) had better prognoses (Table 2).

**Figure 4:**
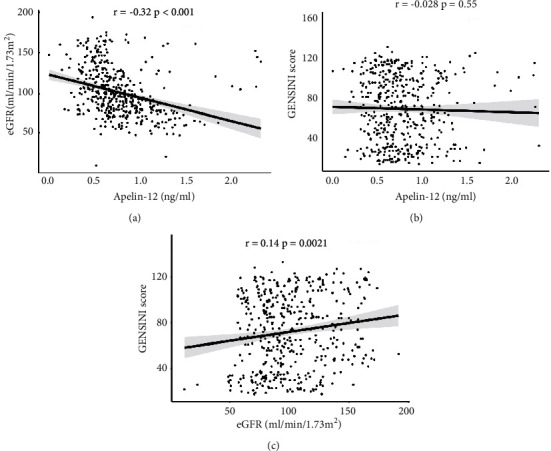
Relationships between apelin-12 or eGFR and Gensini score. (a) Relationship between apelin-12 and eGFR. (b) Relationship between apelin-12 and Gensini score. (c) Relationship between eGFR and Gensini score. eGFR: estimated glomerular filtration rate.

The Gensini score is widely used as an indicator of coronary artery stenosis degree in clinical settings. Therefore, scatter plots were generated and Pearson's correlation coefficients were calculated to explore correlations between apelin-12, eGFR, and Gensini score. There was a significant negative correlation between apelin-12 levels and eGFR (*r* = −0.32, *p* < 0.001). There was a weak correlation between the Gensini score and eGFR (*r* = 0.14, *p*=0.0021). However, there was no significant correlation between the Gensini score and apelin-12 (*r* = -0.028, *p*=0.55) (Figure 4).

## 4. Discussion

STEMI is a major threat to human health worldwide, and prognostic factors have long been explored. In this study, we found that both apelin-12 and eGFR were significant factors influencing STEMI prognosis. More importantly, the combination of apelin-12 and eGFR could efficiently enhance the predictive power of long-term MACEs in patients with STEMI.

Apelin-12, one of the most potent active forms of apelin, has been widely studied in the cardiovascular system. It has been reported that the plasma concentration of apelin-12 after acute myocardial infarction (AMI) is significantly lower than that in the control population [15, 16]. Apelin-12 is negatively associated with troponin I levels, high-sensitivity C-reactive protein, neutrophil/lymphocyte ratio, and the rate of MACE, indicating that apelin-12 can serve as a prognostic biomarker for STEMI patients [9, 17]. This is consistent with the results of our study. As shown by the Kaplan–Meier curves, patients with lower apelin-12 levels (<0.76 ng/mL) exhibited a lower survival probability than those with higher apelin-12 levels. In addition, apelin-12 has been confirmed to exert cardioprotective effects in animal models. Pisarenko and his colleagues explored the efficacy of apelin-12 in an ischemia/reperfusion model using hearts isolated from rats. It was suggested that apelin-12 increased coronary flow and contractile and pump function during reperfusion in a dose-dependent manner [18]. The same investigator also reported that injection of apelin-12 led to a reduction in systolic blood pressure and necrosis markers, including creatinine kinase-MB and lactose dehydrogenase [19].

eGFR is a useful indicator of renal function in clinical practice. It is known that AMI patients with renal impairment experience higher MACE rates and have poorer prognoses [20]. Therefore, eGFR is considered to be an independent predictor of short- and long-term outcomes in patients with AMI [21–24]. The results of our study, in accordance with those from previous research, indicated that a lower eGFR was associated with a poorer prognosis. More importantly, the additive prognostic value of apelin-12 in combination with eGFR was confirmed in our study. As shown by the Kaplan–Meier curves, patients with both low eGFR and apelin-12 experienced the worst outcomes. ROC curve analysis revealed that the combination of apelin-12 and eGFR significantly enhanced the discriminative ability for survival compared with either apelin-12 or eGFR alone. Potential explanations for the positive prognostic value of apelin-12 are as follows. First, apelin can reduce mean arterial pressure and mean circulatory filling pressure via a nitric oxide-dependent mechanism, thus alleviating cardiac afterload and preload. Second, apelin has been shown to increase myocardial contractility by enhancing the sensitivity of myofilaments to activator Ca^2+^, leading to augmentation of cardiac output but without developing left ventricular hypertrophy [25]. Third, apelin can directly protect the heart against ischemia/reperfusion injury by enhancing the expression of endothelial nitric oxide synthase and the phosphorylation of ERK1/2 and Akt [26]. The pro-angiogenic effects of apelin may also explain its association with better prognosis in patients who experience STEMI [27].

Our study showed that there was no correlation between apelin-12 and the degree of coronary artery stenosis indicated by the Gensini score. However, a study by Topuz suggested that apelin-12 was negatively correlated with the angiographic severity, as assessed according to the SYNTAX score [16]. This discrepancy may be due to the different sample sizes and methods used to assess the degree of coronary stenosis. In addition, another study reported that apelin-12 levels were not related to duration or anterior location of ischemia [28]; however, further studies are warranted to confirm these results.

This study has several limitations. First, the sample size of this study was relatively small, especially when the sample was divided into four separate groups. Therefore, the generalization of our conclusions needs further studies. Second, the outcome of our study only included MACE; other endpoints, such as all-cause mortality or cardiac mortality, were not analyzed because detailed information was not provided in the public database. Third, many previous studies grouped patients according to the tertile or quartile of eGFR; however, we only divided patients into high and low GFR groups based on the median eGFR values. Therefore, we were unable to analyze the relationship between the severity of renal impairment and long-term outcomes.

## 5. Conclusions

In summary, our study is the first to suggest that both low apelin-12 levels and low eGFR are associated with poor prognosis in patients with STEMI. The combined use of these two markers can better predict prognosis and guide personalized treatment for STEMI patients.

## Figures and Tables

**Table 1:** Characteristics of patients.

	All patients (*N* = 460)	A (*N* = 70)	B (*N* = 154)	C (*N* = 158)	D (*N* = 78)	*p* value
Age	62.9 ± 11.9	66.3 ± 11.1	59.3 ± 12.1	66.4 ± 11.1	59.9 ± 11.1	<0.001
Sex						<0.001
Male	353 (76.7%)	48 (68.6%)	137 (89.0%)	99 (62.7%)	69 (88.5%)	
Female	107 (23.3%)	22 (31.4%)	17 (11.0%)	59 (37.3%)	9 (11.5%)	
HR	76.9 ± 17.2	78.7 ± 18.2	77.4 ± 16.0	75.9 ± 18.6	76.6 ± 15.3	0.694
SBP	132 ± 27.2	134 ± 28.8	131 ± 27.8	131 ± 27.0	134 ± 24.7	0.693
Anterior wall MI						0.129
No	231 (50.2%)	29 (41.4%)	74 (48.1%)	81 (51.3%)	47 (60.3%)	
Yes	229 (49.8%)	41 (58.6%)	80 (51.9%)	77 (48.7%)	31 (39.7%)	
Killip grade						0.165
I	348 (75.7%)	46 (65.7%)	122 (79.2%)	122 (77.2%)	58 (74.4%)	
≥II	112 (24.3%)	24 (34.3%)	32 (20.8%)	36 (22.8%)	20 (25.6%)	
DM						0.913
No	312 (67.8%)	48 (68.6%)	103 (66.9%)	110 (69.6%)	51 (65.4%)	
Yes	148 (32.2%)	22 (31.4%)	51 (33.1%)	48 (30.4%)	27 (34.6%)	
HTN						0.754
No	196 (42.6%)	33 (47.1%)	61 (39.6%)	68 (43.0%)	34 (43.6%)	
Yes	264 (57.4%)	37 (52.9%)	93 (60.4%)	90 (57.0%)	44 (56.4%)	
Previous MI						0.904
No	405 (88.0%)	61 (87.1%)	137 (89.0%)	140 (88.6%)	67 (85.9%)	
Yes	55 (12.0%)	9 (12.9%)	17 (11.0%)	18 (11.4%)	11 (14.1%)	
Apelin-12	0.76 (0.60–1.00)	0.64 (0.56–0.71)	0.58 (0.48–0.66)	1.03 (0.90–1.22)	0.88 (0.82–1.06)	<0.001
WBC	10.1 ± 3.65	9.96 ± 4.19	10.3 ± 3.74	9.68 ± 3.45	10.5 ± 3.27	0.265
Neutrophils	75.7 ± 11.5	77.4 ± 11.2	74.8 ± 11.8	76.3 ± 11.7	74.7 ± 11.0	0.310
Hemoglobin	144 ± 17.2	145 ± 17.7	144 ± 17.1	143 ± 17.9	143 ± 15.7	0.848
Platelet	232 ± 56.3	239 ± 57.2	230 ± 56.1	230 ± 55.4	234 ± 58.0	0.642
Albumin	38.0 (35.0–41.0)	39.0 (35.0–41.2)	38.0 (35.0–40.9)	38.0 (35.1–40.6)	38.0 (34.0–40.8)	0.636
TC	5.66 ± 1.09	5.91 ± 1.22	5.67 ± 0.99	5.58 ± 1.13	5.62 ± 1.08	0.186
TG	0.99 (0.55–1.54)	0.98 (0.52–1.54)	1.10 (0.55–1.75)	0.90 (0.55–1.39)	0.93 (0.58–1.55)	0.441
HDL	1.20 ± 0.27	1.24 ± 0.30	1.20 ± 0.26	1.19 ± 0.28	1.21 ± 0.24	0.532
LDL	3.00 (2.49–3.60)	3.22 (2.59–3.84)	3.01 (2.50–3.63)	2.81 (2.40–3.38)	3.18 (2.41–3.89)	0.009
FBG	7.67 ± 2.54	7.36 ± 2.38	8.02 ± 2.66	7.51 ± 2.49	7.59 ± 2.47	0.196
Urea nitrogen	6.73 ± 2.07	6.58 ± 2.08	6.65 ± 2.04	6.96 ± 2.00	6.54 ± 2.26	0.378
Creatinine	75.0 (62.0–86.0)	86.0 (79.2–90.2)	59.0 (52.0–69.0)	85.6 (80.0–92.5)	65.9 (58.2–71.8)	<0.001
Uric acid	337 ± 73.9	355 ± 81.6	330 ± 76.2	342 ± 69.7	325 ± 67.4	0.044
eGFR	94.1 (77.8–119)	79.2 (72.4–85.3)	125 (106–145)	76.9 (65.3–84.0)	110 (101–125)	<0.001
CK-MB	106 (45.8–195)	94.5 (42.0–172)	106 (48.0–212)	108 (50.0–190)	132 (38.2–194)	0.668
cTnI	13.9 (4.36–29.0)	13.3 (6.04–29.5)	15.5 (5.01–30.0)	13.9 (3.50–27.3)	12.7 (2.98–27.4)	0.701
Pathological Q wave						0.080
No	239 (52.0%)	34 (48.6%)	75 (48.7%)	95 (60.1%)	35 (44.9%)	
Yes	221 (48.0%)	36 (51.4%)	79 (51.3%)	63 (39.9%)	43 (55.1%)	
Gensini score	72.1 ± 32.2	73.0 ± 33.7	74.5 ± 32.0	66.9 ± 31.4	77.2 ± 31.8	0.069
Culprit vessels						0.029
LAD	230 (50.0%)	42 (60.0%)	76 (49.4%)	80 (50.6%)	32 (41.0%)	
LCX	72 (15.7%)	9 (12.9%)	30 (19.5%)	15 (9.49%)	18 (23.1%)	
RCA	158 (34.3%)	19 (27.1%)	48 (31.2%)	63 (39.9%)	28 (35.9%)	
Stent number	1.38 ± 0.54	1.37 ± 0.62	1.32 ± 0.50	1.43 ± 0.57	1.38 (0.52)	0.399

**Table 2:** Univariate and multivariate Cox regression.

Variables	Univariate Cox			Multivariate Cox		
	HR	95% CI	*p*	HR	95% CI	*p*
Age	1.04	1.02–1.06	<0.001	1.03	1.01–1.05	8.00E-04
Sex						
Female vs. male	1.27	0.85–1.90	0.240			
HR	1.01	1.00–1.02	0.043	1.00	0.99–1.01	0.4481
SBP	1.00	0.99–1.01	0.809			
Anterior wall MI						
Yes vs. no	1.71	1.18–2.47	0.005	1.57	1.06–2.32	0.0243
Killip grade						
II vs. I	1.89	1.29–2.76	0.001	1.49	0.99–2.24	0.0557
DM						
Yes vs. no	1.12	0.77–1.63	0.562			
HTN						
Yes vs. no	1.18	0.81–1.70	0.391			
Previous MI						
Yes vs. no	1.53	0.93–2.49	0.091			
Apelin-12	0.22	0.11–0.44	<0.001			
WBC	1.03	0.98–1.09	0.183			
Neutrophils	1.01	0.99–1.03	0.203			
Hemoglobin	0.98	0.97–0.99	0.001	0.99	0.98–1.00	0.0944
Platelet	1.00	1.00–1.01	0.080			
Albumin	1.00	0.95–1.04	0.853			
TC	1.20	1.04–1.40	0.014	1.23	1.05–1.44	0.0121
TG	0.92	0.73–1.16	0.467			
HDL	2.11	1.07–4.17	0.031	1.06	0.53–2.11	0.8639
LDL	1.06	0.83–1.35	0.659			
FBG	1.00	0.93–1.07	0.922			
Urea nitrogen	1.01	0.93–1.10	0.832			
Creatinine	1.00	1.00–1.01	0.31			
Uric acid	1.00	1.00–1.02	0.647			
eGFR	0.99	0.98–1.00	0.004			
CK-MB	1.00	1.00–1.03	0.475			
cTnI	1.01	1.00–1.03	0.092			
Pathological Q wave						
Yes vs. no	1.72	1.19–2.48	0.004	1.73	1.18–2.53	0.0047
Gensini score	1.00	1.00–1.01	0.141			
Culprit vessels						
LAD	ref	ref	ref			
LCX	0.88	0.52–1.48	0.621			
RCA	0.80	0.53–1.20	0.281			
Stent number	0.88	0.62–1.25	0.472			
Group						
Group A	Ref	Ref	Ref	Ref	Ref	Ref
Group B	0.49	0.30–0.79	0.003	0.6	0.37–0.98	0.0399
Group C	0.51	0.32–0.83	0.006	0.55	0.34–0.89	0.0151
Group D	0.24	0.12–0.49	<0.001	0.27	0.13–0.57	5.00E-04

## Data Availability

The data used in this study can be downloaded from the Dryad database (https://datadryad.org/) using the reference number doi.org/10.5061/dryad.pf56m. Clinical data of 464 patients with STEMI were downloaded from Dryad (https://doi.org/10.5061/dryad.pf56m), a publicly available database that houses a large quantity of datasets from published articles. After removing the data of four patients with missing information, this study ultimately included 460 patients with STEMI from the First People's Hospital of Taizhou, Zhejiang, China, who were admitted with STEMI symptoms between January 2010 and October 2014.

## Abbreviations

AMI: Acute myocardial infarction
AUC: Area under the ROC curve
CI: Confidence interval
eGFR: Estimated glomerular filtration rate
HR: Hazard ratio
MACEs: Major adverse cardiac events
PCI: Percutaneous coronary intervention
ROC: Receiver operating characteristic
STEMI: ST-segment elevation myocardial infarction.
